# Supplementary material for: DHX36 binding induces RNA structurome remodeling and regulates RNA abundance via m6A reader YTHDF1
Source: Nat Commun. 2024 Nov 15;15:9890. doi: 10.1038/s41467-024-54000-y (PMC11564809; doi:10.1038/s41467-024-54000-y)
Supplement: Supplementary file 1 — Supplementary Information [file 41467_2024_54000_MOESM1_ESM.pdf]

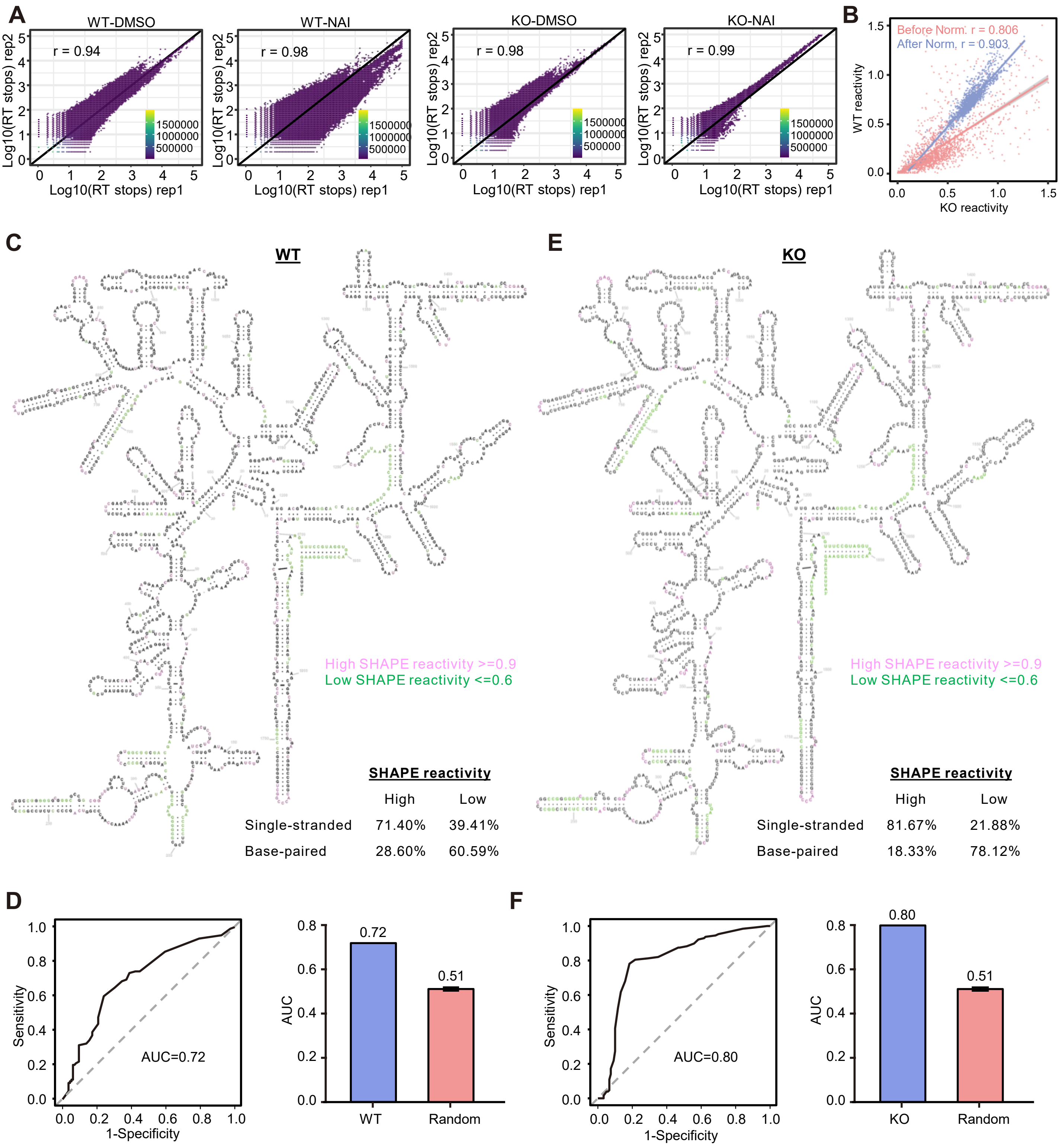
A
WT-DMSO
r = 0.94
WT-NAI
r = 0.98
KO-DMSO
r = 0.98
KO-NAI
r = 0.99
Log10(RT stops) rep2
Log10(RT stops) rep1
1500000
1000000
500000
B
Before Norm: r = 0.806
After Norm, r = 0.903
WT reactivity
KO reactivity
C
WT
High SHAPE reactivity >=0.9
Low SHAPE reactivity <=0.6
SHAPE reactivity
High Low
Single-stranded 71.40% 39.41%
Base-paired 28.60% 60.59%
E
KO
High SHAPE reactivity >=0.9
Low SHAPE reactivity <=0.6
SHAPE reactivity
High Low
Single-stranded 81.67% 21.88%
Base-paired 18.33% 78.12%
D
Sensitivity
1-Specificity
AUC=0.72
AUC
0.72
0.51
WT
Random
F
Sensitivity
1-Specificity
AUC=0.80
AUC
0.80
0.51
KO
Random

Supplementary Fig. 1 *In vivo* RNA structurome profiling unveils DHX36 depletion-induced global increase of RNA structures. (A) Scatterplot showing the correlation between RT stops in two biological replicates. r represents Pearson correlation coefficient. (B) Scatterplot showing the correlations between the SHAPE reactivities of 18S rRNA in WT and KO samples. The colors of scatters and lines represent the status of normalization. r represents the correlation coefficient calculated by Pearson correlation test. (C) Structure-seq strategy in WT HEK293T cells was benchmarked by 18S rRNA structure at single-nucleotide resolution. 71.40% (true positive) of the bases with high SHAPE reactivity (>=0.9) correspond to single-stranded regions in the phylogenetic structure of 18S rRNA. 60.59% (true negative) of the bases with low SHAPE reactivity (<=0.6) correspond to base paired regions in the phylogenetic structure. (D) Left: receiver operating characteristic (ROC) curve showing the agreement between the reactivity scores of WT Structure-seq data and the phylogenetic structure of 18S rRNA. Right: histograms showing the comparison of the area under curve (AUC) scores between true reactivity scores of the WT sample (n=1 true set) and the randomly shuffled reactivity scores (n=100 random sets). (E) Structure-seq strategy in DHX36-KO HEK293T cells was benchmarked by 18S rRNA structure at single-nucleotide resolution. 81.67% (true positive) of the bases with high SHAPE reactivity (>=0.9) correspond to single-stranded regions in the phylogenetic structure of 18S rRNA. 78.12% (true negative) of the bases with low SHAPE reactivity (<=0.6) correspond to base paired regions in the phylogenetic structure. (F) Left: ROC curves showing the agreement between the reactivity scores of DHX36-KO Structure-seq data and the phylogenetic structure of 18S rRNA. Right: histograms showing the comparison of the AUC scores between true reactivity scores of the DHX36-KO sample (n=1 true set) and the randomly shuffled reactivity scores (n=100 random sets). Data are presented as mean values +/- SD in (D and F).

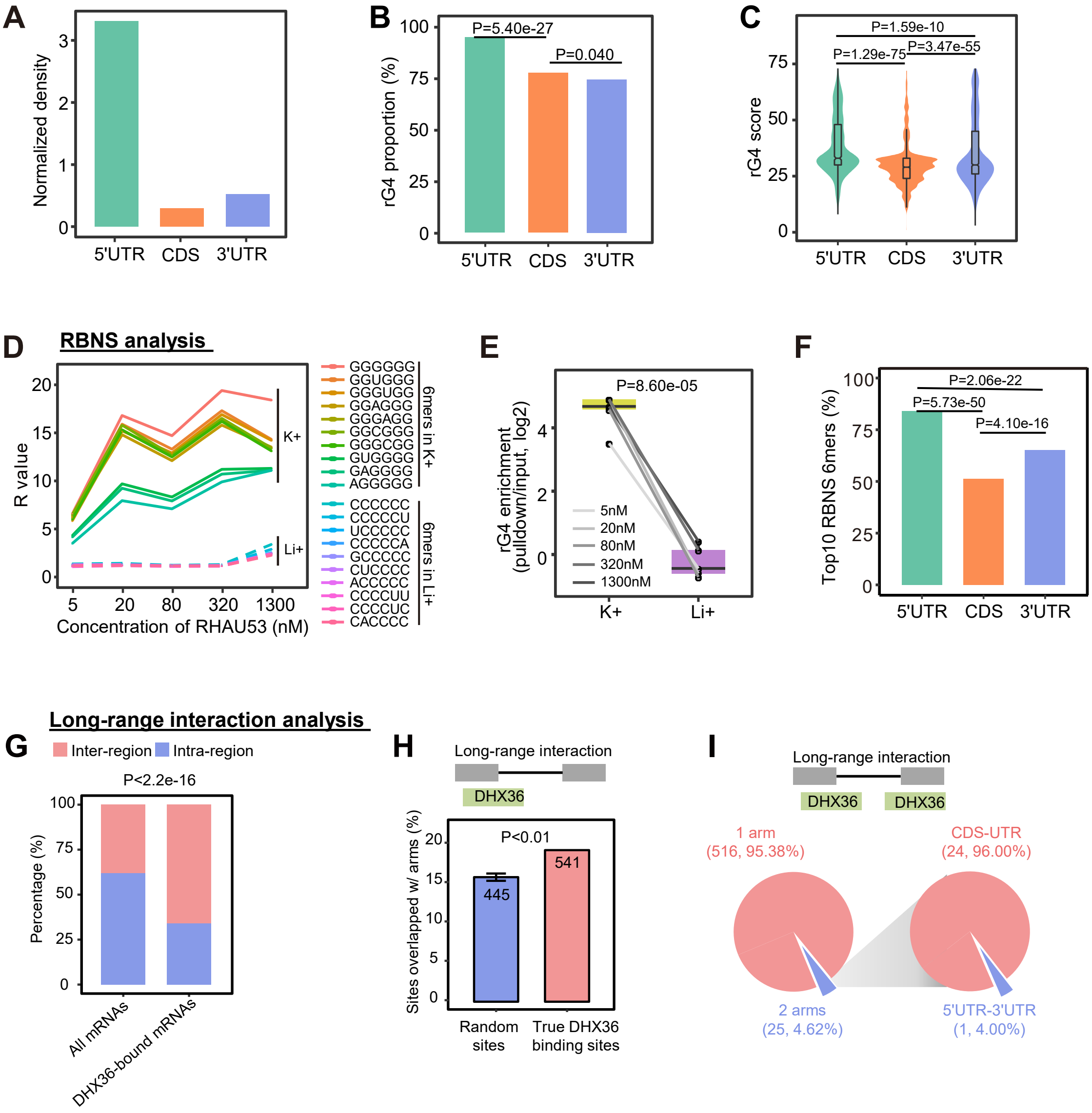

A
Normalized density
5'UTR
CDS
3'UTR
B
rG4 proportion (%)
P=5.40e-27
P=0.040
C
rG4 score
P=1.59e-10
P=1.29e-75
P=3.47e-55
D
RBNS analysis
R value
Concentration of RHAU53 (nM)
GGGGGG
GGUGGG
GGGUGG
GGAGGG
GGGAGG
GGCGGG
GGGCGG
GUGGGG
GAGGGG
AGGGGG
6mers in K+
CCCCCC
CCCCCU
UCCCCC
CCCCCA
GCCCCC
CUCCCC
ACCCCC
CCCCUU
CCCCUC
CACCCC
6mers in Li+
K+
Li+
E
rG4 enrichment (pulldown/input, log2)
P=8.60e-05
5nM
20nM
80nM
320nM
1300nM
F
Top10 RBNS 6mers (%)
P=2.06e-22
P=5.73e-50
P=4.10e-16
G
Long-range interaction analysis
Inter-region
Intra-region
P<2.2e-16
Percentage (%)
All mRNAs
DHX36-bound mRNAs
H
Long-range interaction
DHX36
Sites overlapped w/ arms (%)
P<0.01
445
541
Random sites
True DHX36 binding sites
I
Long-range interaction
DHX36
DHX36
1 arm
(516, 95.38%)
2 arms
(25, 4.62%)
CDS-UTR
(24, 96.00%)
5'UTR-3'UTR
(1, 4.00%)

Supplementary Fig. 2 PAR-CLIP and RBNS data analyses reveal binding preference of DHX36. (A) The binding density of DHX36 in the designated mRNA regions. The normalized density is defined as the number of binding sites *1000 divided by the total length of the designated region. (B) The proportions of rG4-containing DHX36 binding sites in the designated regions of mRNAs. Statistical significance was calculated by Chi-square test. (C) Comparison of rG4 scores among the rG4s located in different mRNA regions. Statistical significance was calculated by unpaired two-sided Student's t-test. The boxes indicate median (centre), Q25 and Q75 (bounds of box), the smallest value within 1.5 times interquatile range below Q25 and largest value within 1.5 times interquatile range above Q75 (whiskers). (D) The distributions of R values of top 10 enriched 6mer motifs bound by RHAU53 peptides in the presence of K+ or Li+. (E) Comparison of rG4 enrichment within RHAU53-bound RNAs between K+ and Li+ conditions. Statistical significance was calculated by paired two-sided Student's t-test. (F) The percentage of DHX36 binding sites within the designated mRNA regions covered by top10 enriched K+ RBNS 6mer motifs. Statistical significance was calculated by Chi-square test. (G) Comparison of the percentage of the genes with inter-region interactions between DHX36-bound mRNAs and all mRNAs. Statistical significance was calculated by Chi-square test. (H) Comparison of the sites overlapped with at least one arm of long-range interactions between true DHX36 binding sites (n=1 true set) and 100 random sets (n=100 random sets). Each random set was composed of the randomly selected sites in equal numbers, equal length, and from the same mRNA region. Statistical significance was calculated by Monte Carlo method. (I) Left: pie chart showing the proportion of the DHX36-bound mRNAs with binding sites overlapped with both arms of long-range interaction. Right: pie chart showing the proportion of 5'UTR-3'UTR and CDS-UTR interactions. At the top panel of (H) and (I), the grey and green blocks represent the arms of long-range interactions and true DHX36/random sites, respectively.

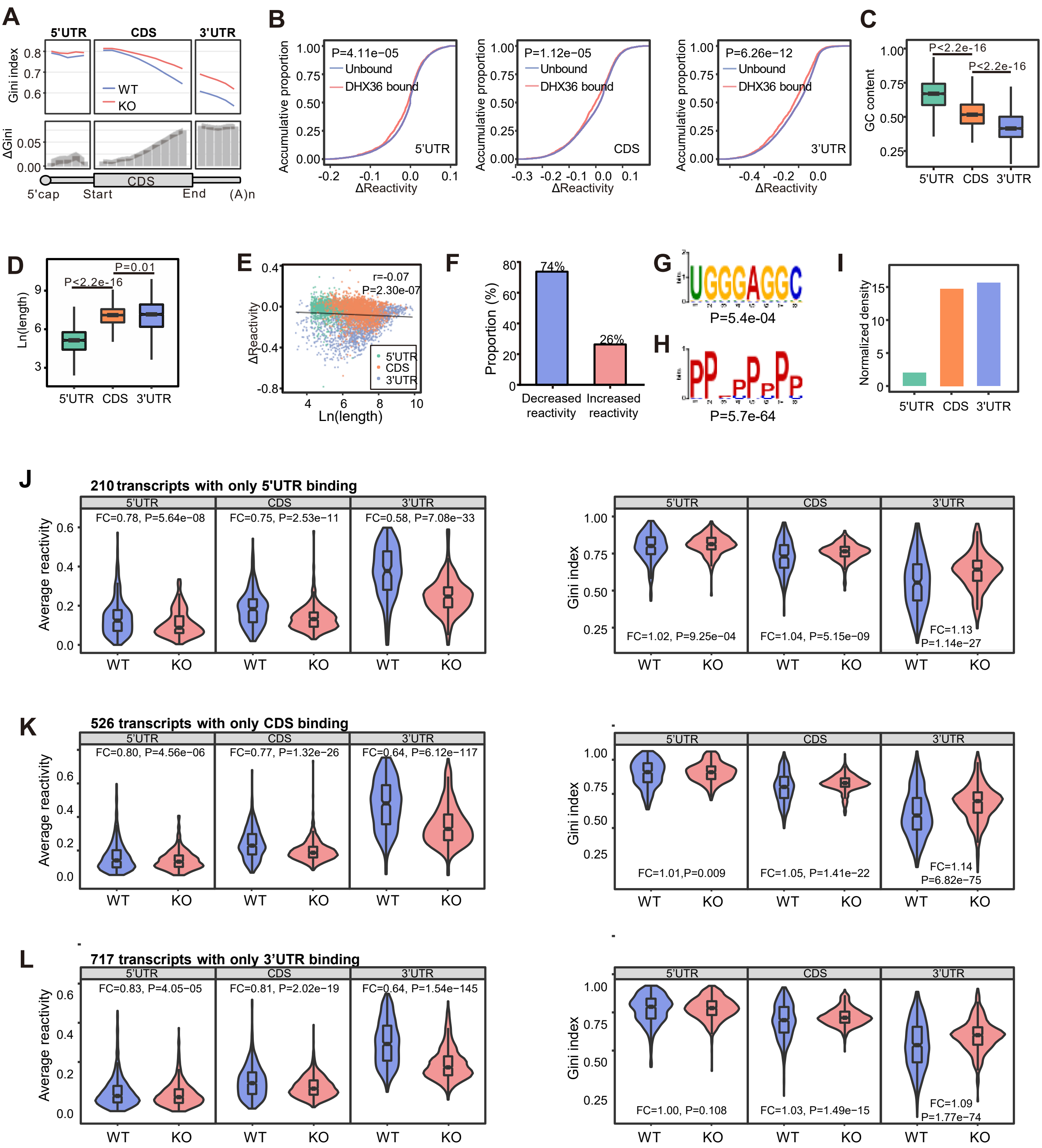
A
5'UTR
CDS
3'UTR
Gini index
WT
KO
ΔGini
5'cap
Start
End
(A)n
B
P=4.11e−05
Unbound
DHX36 bound
5'UTR
Accumulative proportion
ΔReactivity
P=1.12e−05
CDS
P=6.26e−12
3'UTR
C
P<2.2e-16
GC content
D
P=0.01
Ln(length)
E
r=-0.07
P=2.30e-07
ΔReactivity
Ln(length)
F
74%
26%
Proportion (%)
Decreased reactivity
Increased reactivity
G
UGGGAGGC
P=5.4e-04
H
P=5.7e-64
I
Normalized density
J
210 transcripts with only 5'UTR binding
FC=0.78, P=5.64e−08
FC=0.75, P=2.53e−11
FC=0.58, P=7.08e−33
Average reactivity
FC=1.02, P=9.25e−04
FC=1.04, P=5.15e−09
FC=1.13
P=1.14e−27
Gini index
K
526 transcripts with only CDS binding
FC=0.80, P=4.56e−06
FC=0.77, P=1.32e−26
FC=0.64, P=6.12e−117
FC=1.01,P=0.009
FC=1.05, P=1.41e−22
FC=1.14
P=6.82e−75
L
717 transcripts with only 3'UTR binding
FC=0.83, P=4.05−05
FC=0.81, P=2.02e−19
FC=0.64, P=1.54e−145
FC=1.00, P=0.108
FC=1.03, P=1.49e−15
FC=1.09
P=1.77e−74

Supplementary Fig. 3 DHX36 induces localized structural loss of 3’UTR binding sites. (A) Top: the binned average Gini index across the length of 5’UTRs (5 bins), CDSs (10 bins) and 3’UTRs (5 bins) of DHX36-bound mRNAs. Bottom: The binned ΔGini (KO-WT) across the above regions. Shaded area represents 95% confidence intervals of the average ΔGini of each bin calculated by paired two-sided Student’s t-test. (B) Comparison of ΔReactivity (KO-WT) of 5’UTR, CDS, and 3’UTR between DHX36-bound and unbound mRNAs. Two-sided Wilcoxon rank-sum test was used to calculate the significance. (C) GC content and (D) length of the designated region of DHX36-bound mRNAs (n=1787). (E) Scatterplot showing regional ΔReactivity vs. length of the designated regions of DHX36-bound mRNAs. r and P represent the correlation coefficient and statistical significance calculated by Pearson correlation test. (F) 74% of DRRs within DHX36-bound mRNAs showed decreased reactivity upon DHX36 loss. (G) The enriched sequence motif of DRRs with the highest significance. (H) The enriched differential structural motif of DRRs in the KO vs. WT cells with the highest significance. “P” and “U” denote “paired” and “unpaired” structures, respectively. (I) The density of DRRs in the designated mRNA regions, upon normalization by DHX36 binding frequency. (J-L) The average reactivity (left) and Gini index (right) of reactivity scores of the designated mRNA region with DHX36 binding events only in (J) 5’UTR (n=210), (K) CDS (n=526) and (L) 3’UTR (n=717). Statistical significance was calculated by two-sided Wilcoxon signed-rank test. The boxes in this figure indicate median (centre), Q25 and Q75 (bounds of box), the smallest value within 1.5 times interquatile range below Q25 and largest value within 1.5 times interquatile range above Q75 (whiskers). Two-sided Wilcoxon rank-sum test was used to calculate the significance.

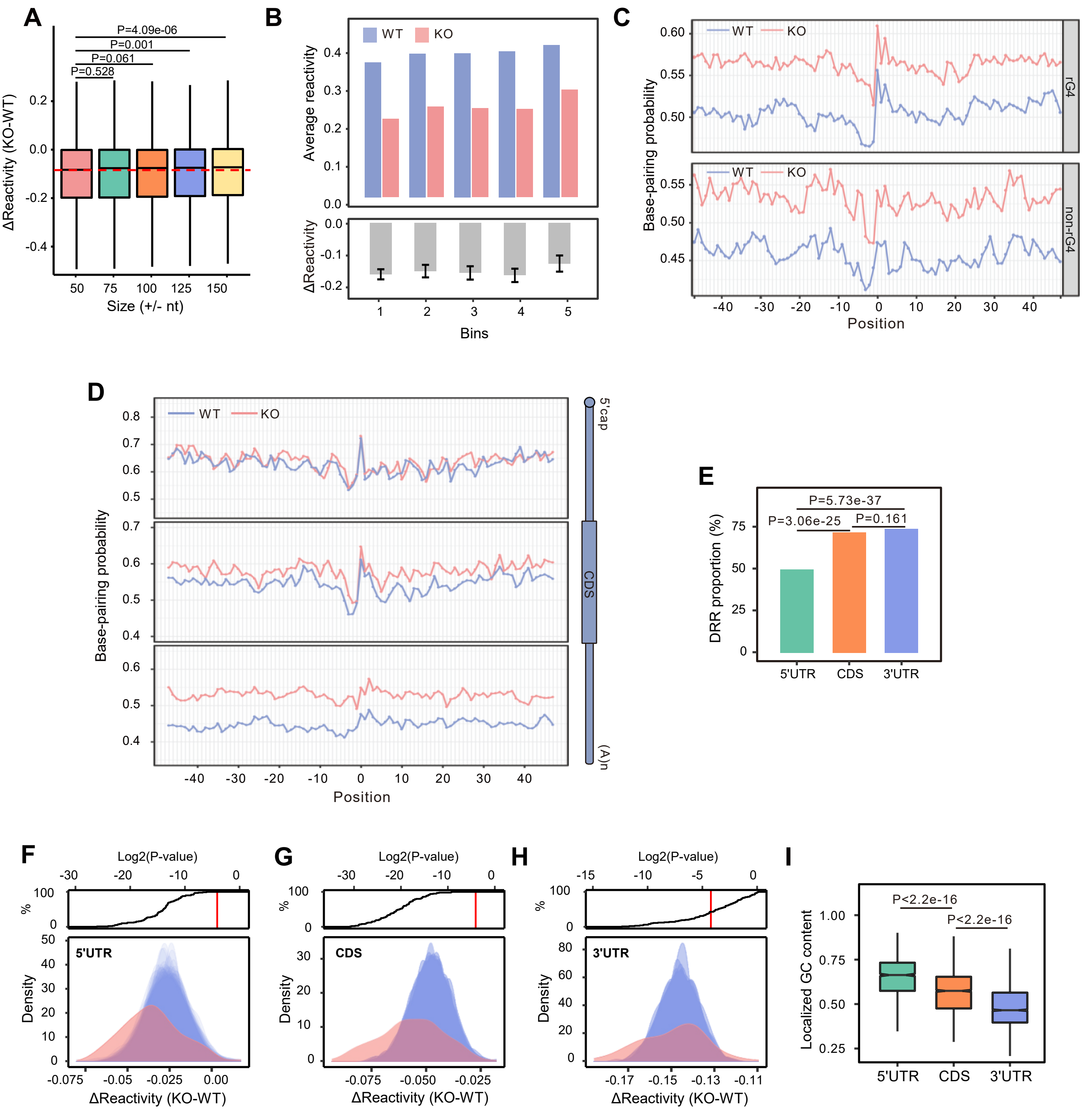

A
P=4.09e-06
P=0.001
P=0.061
P=0.528
ΔReactivity (KO-WT)
Size (+/- nt)
B
WT
KO
Average reactivity
ΔReactivity
Bins
C
WT
KO
rG4
non-rG4
Base-pairing probability
Position
D
WT
KO
5'cap
CDS
(A)n
Base-pairing probability
Position
E
P=5.73e-37
P=3.06e-25
P=0.161
DRR proportion (%)
5'UTR
CDS
3'UTR
F
Log2(P-value)
5'UTR
Density
ΔReactivity (KO-WT)
G
Log2(P-value)
CDS
Density
ΔReactivity (KO-WT)
H
Log2(P-value)
3'UTR
Density
ΔReactivity (KO-WT)
I
P<2.2e-16
P<2.2e-16
Localized GC content
5'UTR
CDS
3'UTR

Supplementary Fig. 4 DHX36 induces localized structural loss of 3'UTR binding sites. (A) Comparison of the average ΔReactivity of the sites of various sizes (n=4263). Statistical significance was calculated by two-sided Wilcoxon signed-rank test. (B) The binned average reactivity (top) and ΔReactivity (bottom) of DHX36-bound DRRs. Error bars in the bottom panel represent 95% confidence intervals (CI) of the average ΔReactivity of each bin. (C) BPP across DHX36 binding sites with and without rG4s. (D) Comparison of BPP across DHX36 binding sites located in 5'UTRs (top), CDSs (middle) and 3'UTRs (bottom). (E) The percentage of DRR-containing binding sites in the designated mRNA regions. Statistical significance was calculated by Chi-square test. (F-H) Comparison of the ΔReactivities between 100 random sets (purple) and the true DHX36 binding sites (pink) within (F) 5'UTR, (G) CDS, and (H) 3'UTR. Each random set was composed of the randomly selected sites in equal numbers, equal length, and from the same mRNA region, and was compared to true DHX36 binding sites to generate a p-value. The distribution density of ΔReactivities and the accumulative proportion (%) of the log2(P-values) were shown in the bottom and top panels, respectively. (I) Localized GC content of DHX36 binding sites in the designated mRNA region. Statistical significance was calculated by two-sided Wilcoxon rank-sum test.

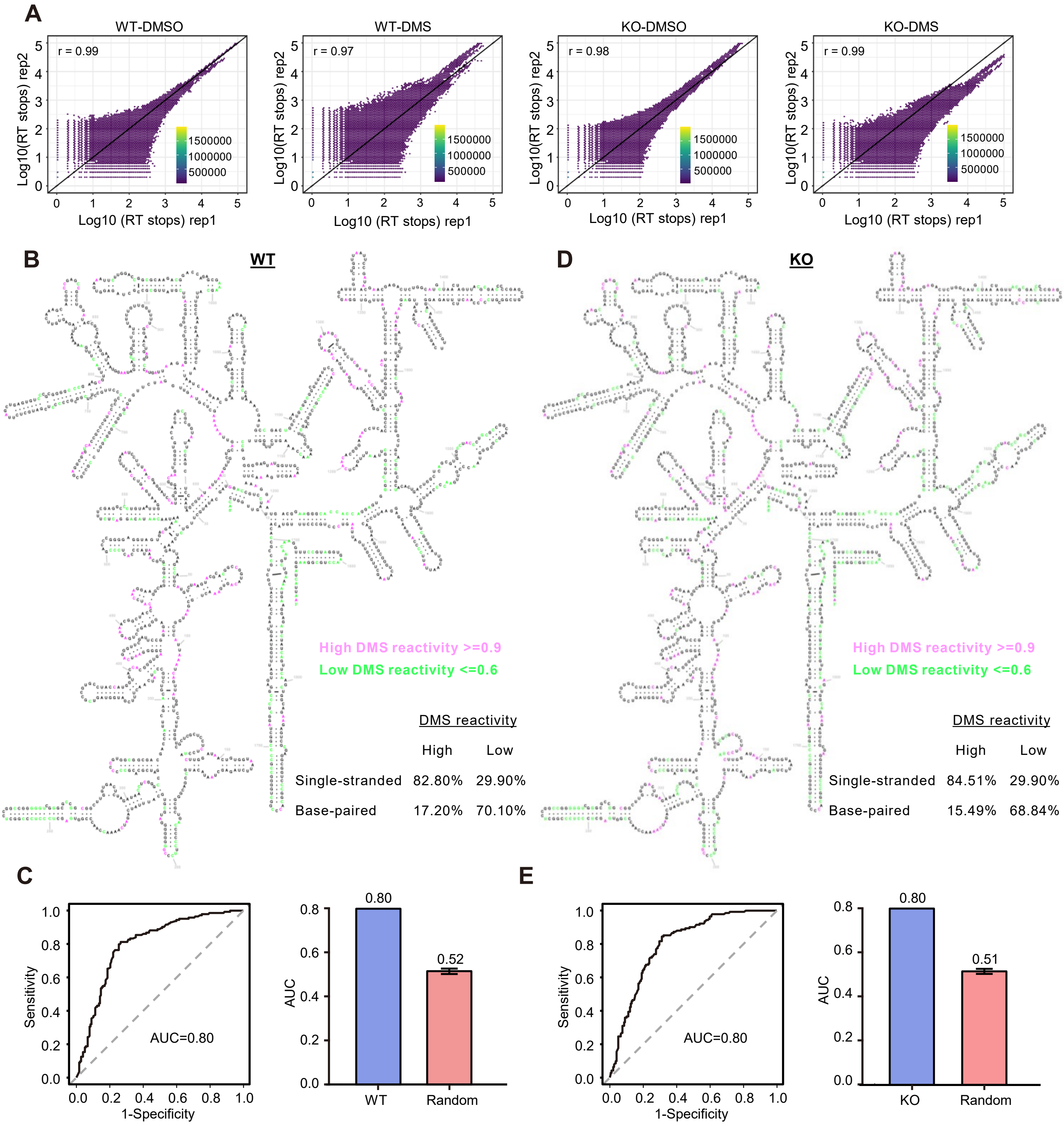
A
WT-DMSO
r = 0.99
WT-DMS
r = 0.97
KO-DMSO
r = 0.98
KO-DMS
r = 0.99
Log10(RT stops) rep2
Log10 (RT stops) rep1
1500000
1000000
500000
B
WT
D
KO
High DMS reactivity >=0.9
Low DMS reactivity <=0.6
DMS reactivity
High Low
Single-stranded 82.80% 29.90%
Base-paired 17.20% 70.10%
Single-stranded 84.51% 29.90%
Base-paired 15.49% 68.84%
C
E
Sensitivity
1-Specificity
AUC=0.80
AUC
0.80
0.52
0.51
WT
KO
Random

Supplementary Fig. 5 DMS Structure-seq libraries in WT and Dhx36-KO C2C12 myoblast cells. (A) Scatterplot showing the correlation between RT stops in biological replicates 1 vs 2. r represents Pearson correlation coefficient. (B) Structure-seq strategy in WT C2C12 cells was benchmarked by 18S rRNA structure at single-nucleotide resolution. 82.80% (true positive) of the bases with high DMS reactivity (>=0.9) correspond to single-stranded regions in the phylogenetic structure of 18S rRNA. 70.10% (true negative) of the bases with low DMS reactivity (<=0.6) correspond to base paired regions in the phylogenetic structure. (C) Left: ROC curve showing the agreement between the reactivity scores of WT Structure-seq data and the phylogenetic structure of 18S rRNA. Right: histograms showing the comparison of the AUC scores between true reactivity scores of the WT sample (n=1 true set) and the randomly shuffled reactivity scores (n=100 random sets). (D) Structure-seq strategy in Dhx36-KO C2C12 cells was benchmarked by 18S rRNA structure at single-nucleotide resolution. 84.51% (true positive) of the bases with high DMS reactivity (>=0.9) correspond to single-stranded regions in the phylogenetic structure of 18S rRNA. 68.84% (true negative) of the bases with low DMS reactivity (<=0.6) correspond to base paired regions in the phylogenetic structure. (E) Left: ROC curves showing the agreement between the reactivity scores of Dhx36-KO Structure-seq data and the phylogenetic structure of 18S rRNA. Right: histograms showing the comparison of the AUC scores between true reactivity scores of the Dhx36-KO sample (n=1 true set) and the randomly shuffled reactivity scores (n=100 random sets). Data are presented as mean values +/- SD in (C and E).

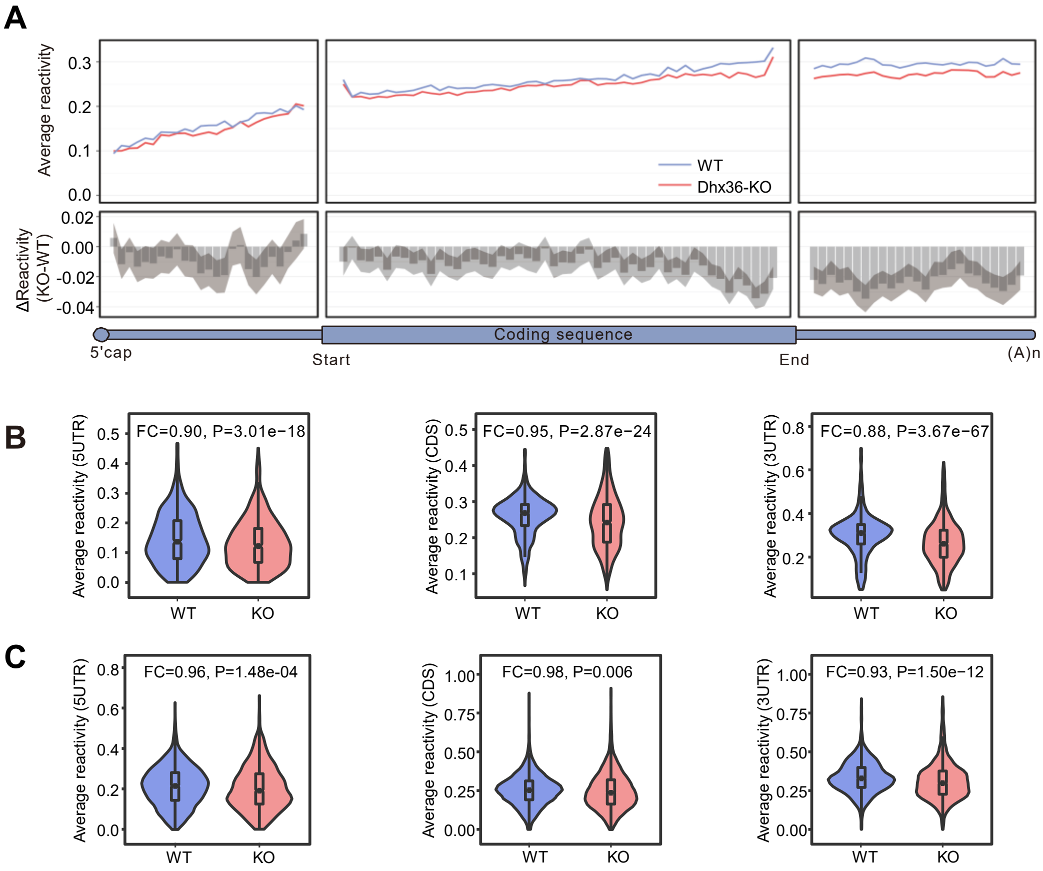

Supplementary Fig. 6 DHX36 binding-induced structure remodeling effect is conserved in C2C12 myoblast cells. (A) Top: the binned average reactivity across the length of 5'UTRs (25 bins), CDSs (50 bins) and 3'UTRs (25 bins) of DHX36-bound mRNAs. Bottom: the binned ΔReactivity (KO-WT) across the above regions. Shaded area represents 95% confidence intervals of the average ΔReactivity of each bin calculated by paired two-sided Student's t-test. (B) The average reactivity of 5'UTRs, CDS, and 3'UTRs of DHX36-bound mRNAs. Two-sided Wilcoxon signed-rank test was used to calculate the statistical significance. The fold change (FC) between the KO vs. WT is shown. (C) The average reactivity of DHX36 binding sites within 5'UTRs (left), CDS (middle) and 3'UTRs (right). Statistical significance was calculated by two-sided Wilcoxon signed-rank test. The boxes in this figure indicate median (centre), Q25 and Q75 (bounds of box), the smallest value within 1.5 times interquatile range below Q25 and largest value within 1.5 times interquatile range above Q75 (whiskers).

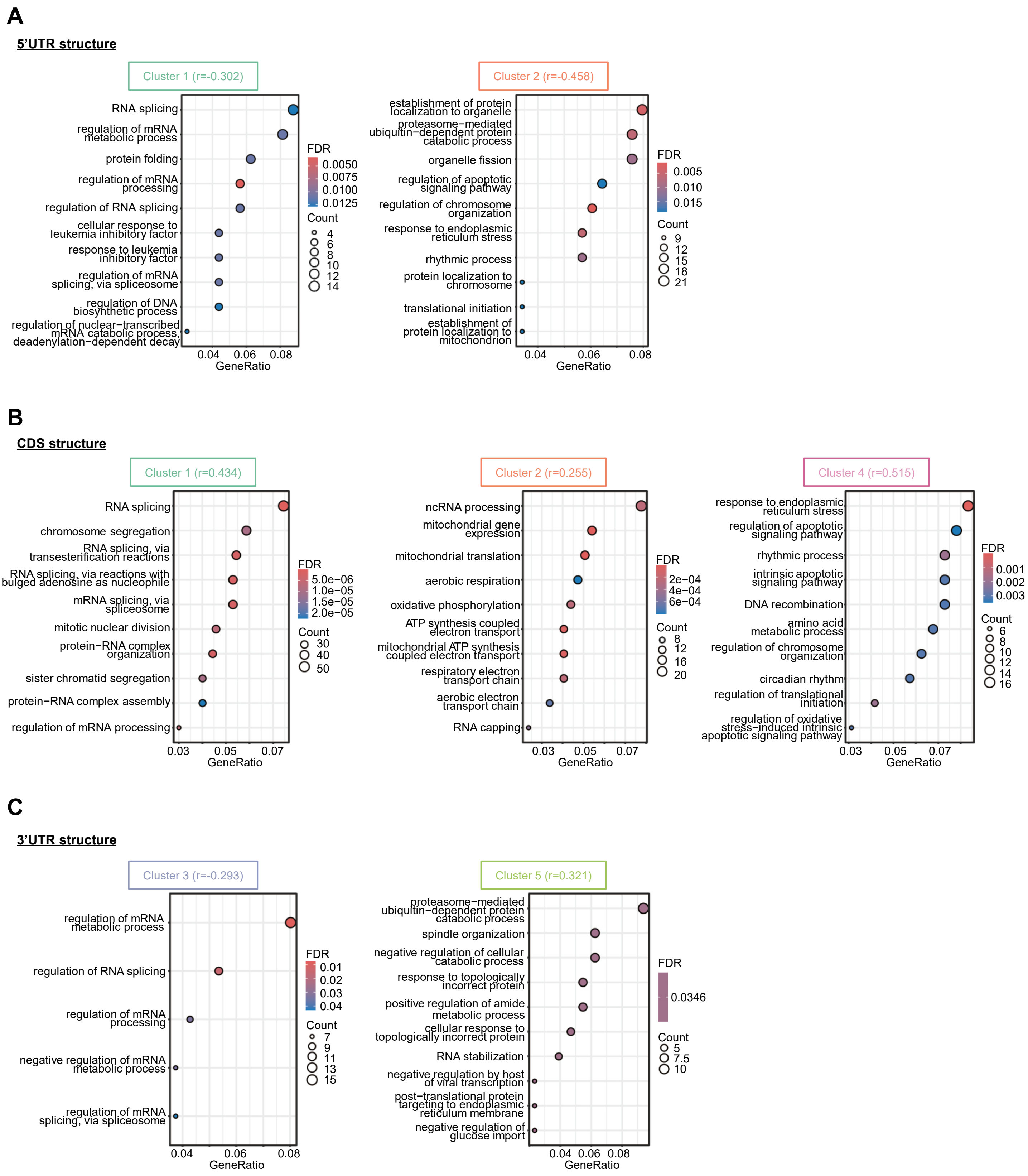

Supplementary Fig. 7 GO enrichment analysis on the subsets of mRNAs most strongly affected by the DHX36-induced structural changes. GO analysis was performed on the mRNA subsets with significant correlations (|r| > 0.25) between mRNA abundance changes and structural changes in their (A) 5'UTR, (B) CDS, and (C) 3'UTR regions. The color and size of dots represent the adjusted P-values and the gene counts annotated to each GO term, respectively. The cluster labels correspond to those in Fig. 4E.

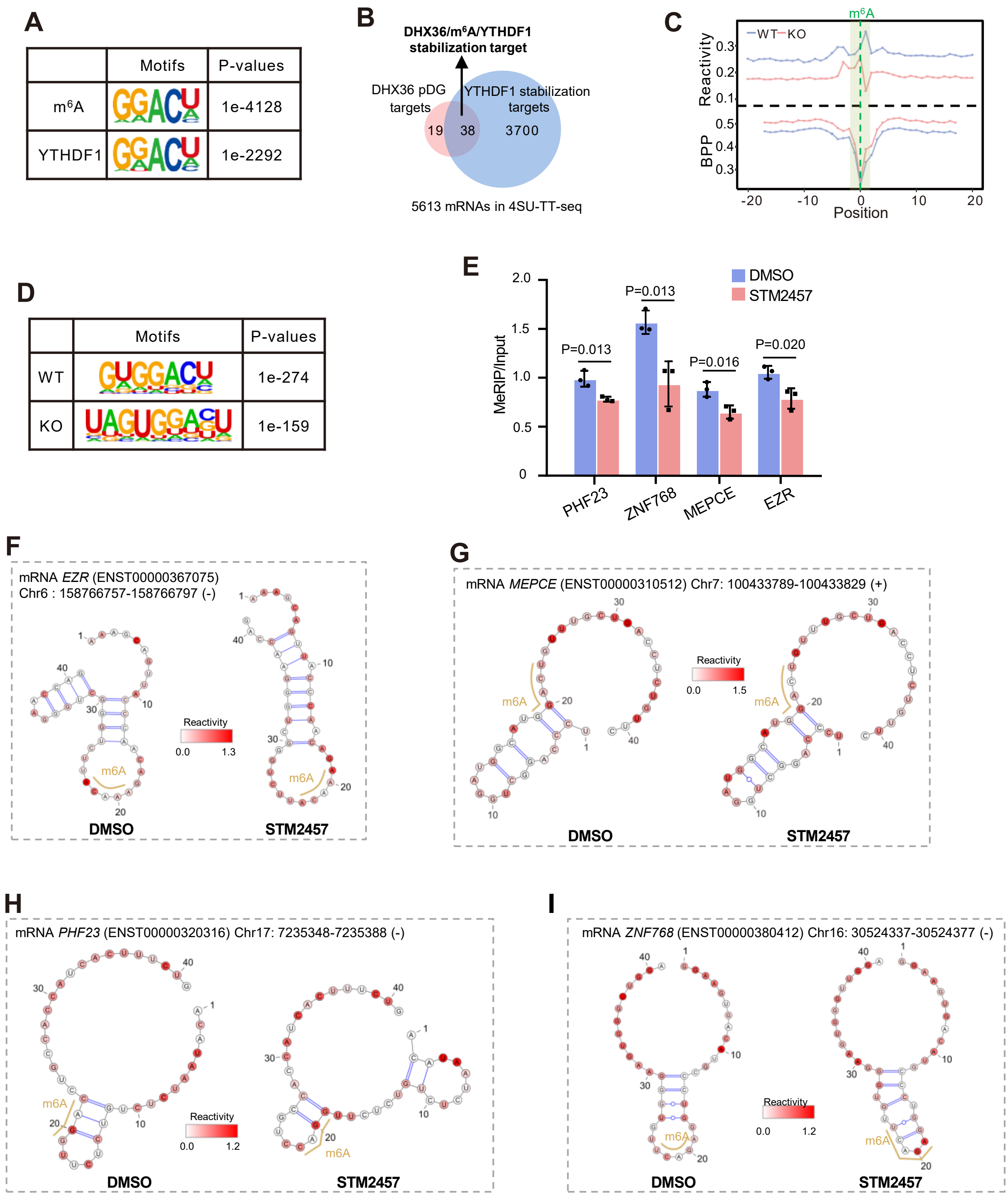
A
Motifs
P-values
m6A
1e-4128
YTHDF1
1e-2292
B
DHX36/m6A/YTHDF1 stabilization target
DHX36 pDG targets
YTHDF1 stabilization targets
19
38
3700
5613 mRNAs in 4SU-TT-seq
C
m6A
WT
KO
Reactivity
BPP
Position
D
Motifs
P-values
WT
1e-274
KO
1e-159
E
DMSO
STM2457
MeRIP/Input
P=0.013
P=0.013
P=0.016
P=0.020
PHF23
ZNF768
MEPCE
EZR
F
mRNA EZR (ENST00000367075)
Chr6 : 158766757-158766797 (-)
Reactivity
DMSO
STM2457
G
mRNA MEPCE (ENST00000310512) Chr7: 100433789-100433829 (+)
Reactivity
DMSO
STM2457
H
mRNA PHF23 (ENST00000320316) Chr17: 7235348-7235388 (-)
Reactivity
DMSO
STM2457
I
mRNA ZNF768 (ENST00000380412) Chr16: 30524337-30524377 (-)
Reactivity
DMSO
STM2457

Supplementary Fig. 8 Transcriptomic m6A and YTHDF1 binding profiles. (A) De novo motif discovery on m6A sites and YTHDF1 binding sites. (B) Venn diagram showing insignificant overlapping between pDGs with DHX36 binding to 3'UTRs and target mRNAs stabilized by YTHDF1. Hypergeometric test was used to calculate the statistical significance. (C) Comparison of average reactivity and BPP of the regions surrounding the transcriptomic m6A sites in WT and DHX36-KO cells. Position 0 and green area denote m6A residues and m6A motif respectively. (D) De novo motif discovery of YTHDF1 binding sites in the YTHDF1 CLIP-seq data performed in WT and DHX36-KO cells. (E) HEK293T cells were treated with STM2457 and MeRIP-qPCR was performed to confirm the decreased m6A levels upon STM2457 treatment. n=3 biological replicates. Bars represent mean ± SD. Statistical significance was determined using a two-sided Student's t- test. (F-I) Illustration of the folded structures of the m6A sites within (F) EZR, (G) MEPCE, (H) PHF23, and (I) ZNF768 in STM2457-treated and control HEK293T cells. m6A sites are highlighted using yellow lines. Nucleotides are color-coded based on the SHAPE reactivity scores.
