# Supplementary material for: DHX36 binding induces RNA structurome remodeling and regulates RNA abundance via m6A reader YTHDF1
Source: Nat Commun. 2024 Nov 15;15:9890. doi: 10.1038/s41467-024-54000-y (PMC11564809; doi:10.1038/s41467-024-54000-y)
Supplement: Supplementary file 2 — Description of Additional Supplementary Files [file 41467_2024_54000_MOESM2_ESM.docx]

**Description of Additional Supplementary Information**

Data 1. Structure-seq data analyses in HEK293T cells.

Data 2. DHX36 binding profiles in HEK293T and C2C12 cells.

Data 3. DHX36-induced DRRs within DHX36-bound mRNAs. The significance was calculated by dStruct. The false positive discovery rate (FDR) method was used to correct for multiple comparisons.

Data 4. Variance inflation factors (VIF) of the explanatory variables in the linear regression models.

Data 5. Structure-seq data analyses in C2C12 cells.

Data 6. Identification of DHX36 post-transcriptional regulatory targets.

Data 7. Identification of mRNA clusters with strong correlation between mRNA abundance and structural changes within the designated regions.

Data 8. m6A and YTHDF1 binding sites within the DHX36 binding sites located in 3UTRs.

Data 9. Sequences of DNA, RNA oligos and peptide used in this study.
