# Supplementary material for: DHX36 binding induces RNA structurome remodeling and regulates RNA abundance via m6A reader YTHDF1
Source: Nat Commun. 2024 Nov 15;15:9890. doi: 10.1038/s41467-024-54000-y (PMC11564809; doi:10.1038/s41467-024-54000-y)
Supplement: Supplementary file 12 — Reporting Summary [file 41467_2024_54000_MOESM12_ESM.pdf]

nature portfolio

Corresponding author(s): Huating Wang

Last updated by author(s): Sep 28, 2024

Reporting Summary

Nature Portfolio wishes to improve the reproducibility of the work that we publish. This form provides structure for consistency and transparency in reporting. For further information on Nature Portfolio policies, see our Editorial Policies and the Editorial Policy Checklist.

Statistics

For all statistical analyses, confirm that the following items are present in the figure legend, table legend, main text, or Methods section.

n/a	Confirmed	
☐	☒	The exact sample size (*n*) for each experimental group/condition, given as a discrete number and unit of measurement
☐	☒	A statement on whether measurements were taken from distinct samples or whether the same sample was measured repeatedly
☐	☒	The statistical test(s) used AND whether they are one- or two-sided<br>*Only common tests should be described solely by name; describe more complex techniques in the Methods section.*
☐	☒	A description of all covariates tested
☐	☒	A description of any assumptions or corrections, such as tests of normality and adjustment for multiple comparisons
☐	☒	A full description of the statistical parameters including central tendency (e.g. means) or other basic estimates (e.g. regression coefficient) AND variation (e.g. standard deviation) or associated estimates of uncertainty (e.g. confidence intervals)
☐	☒	For null hypothesis testing, the test statistic (e.g. *F*, *t*, *r*) with confidence intervals, effect sizes, degrees of freedom and *P* value noted<br>*Give P values as exact values whenever suitable.*
☒	☐	For Bayesian analysis, information on the choice of priors and Markov chain Monte Carlo settings
☐	☒	For hierarchical and complex designs, identification of the appropriate level for tests and full reporting of outcomes
☐	☒	Estimates of effect sizes (e.g. Cohen's *d*, Pearson's *r*), indicating how they were calculated

Our web collection on statistics for biologists contains articles on many of the points above.

Software and code

Policy information about availability of computer code

Data collection	No software was used for data collection.
Data analysis	Cutadapt v1.15; Bowtie2 v2.3.3.1; StructureFold2; BWA v0.7.17; CLIP Tool Kit v1.1.3; Piranha v1.2.1; STAR v2.5.2; HISAT2 v2.0.4; featureCounts v2.0.1; Trimmomatic v0.30; Homer v4.9.1; MEME suite v5.5; ViennaRNA v2.5.1; DESeq2 v1.18; Cufflinks v2.2.1; deepTools v3.5.6; Samtools v1.6

For manuscripts utilizing custom algorithms or software that are central to the research but not yet described in published literature, software must be made available to editors and reviewers. We strongly encourage code deposition in a community repository (e.g. GitHub). See the Nature Portfolio guidelines for submitting code & software for further information.

Data

Policy information about availability of data

All manuscripts must include a data availability statement. This statement should provide the following information, where applicable:

- Accession codes, unique identifiers, or web links for publicly available datasets
- A description of any restrictions on data availability
- For clinical datasets or third party data, please ensure that the statement adheres to our policy

Structure-seq and RBNS data used in this study have been deposited in Gene Expression Omnibus (GEO) database under the accession codes GSE237160 (WT and DHX36/Dhx36 Structure-seq), GSE237161 (RBNS), GSE264498 (YTHDF1 CLIP-seq), and GSE264642 (Control and m6A-inhibited Structure-seq).

Research involving human participants, their data, or biological material

Policy information about studies with human participants or human data. See also policy information about sex, gender (identity/presentation), and sexual orientation and race, ethnicity and racism.

Reporting on sex and gender	Not applicable.
Reporting on race, ethnicity, or other socially relevant groupings	Not applicable.
Population characteristics	Not applicable.
Recruitment	Not applicable.
Ethics oversight	Not applicable.

Note that full information on the approval of the study protocol must also be provided in the manuscript.

Field-specific reporting

Please select the one below that is the best fit for your research. If you are not sure, read the appropriate sections before making your selection.

☒ Life sciences ☐ Behavioural & social sciences ☐ Ecological, evolutionary & environmental sciences

For a reference copy of the document with all sections, see nature.com/documents/nr-reporting-summary-flat.pdf

Life sciences study design

All studies must disclose on these points even when the disclosure is negative.

Sample size	At least two biological replicates per group.
Data exclusions	No data was excluded from
Replication	All experiments were repeated in multiple independent biological replicates.
Randomization	Cells were cultured and he plates/wells were randomly selected.
Blinding	Blinding was not necessary in this study.

Reporting for specific materials, systems and methods

We require information from authors about some types of materials, experimental systems and methods used in many studies. Here, indicate whether each material, system or method listed is relevant to your study. If you are not sure if a list item applies to your research, read the appropriate section before selecting a response.

Materials & experimental systems

n/a	Involved in the study	
☐	☒	Antibodies
☐	☒	Eukaryotic cell lines
☒	☐	Palaeontology and archaeology
☒	☐	Animals and other organisms
☒	☐	Clinical data
☒	☐	Dual use research of concern
☒	☐	Plants

Methods

n/a	Involved in the study	
☒	☐	ChIP-seq
☒	☐	Flow cytometry
☒	☐	MRI-based neuroimaging

Antibodies

Antibodies used	DHX36 (Proteintech 13159-1-AP); YTHDF1 antibody (17479-1-AP, Proteintech); IgG control (2729, Cell Signaling Technology); m6A antibody (Abcam, ab151230); GAPDH (Santa Cruz, sc-32233);

Validation	All antibodies used are commercially available and the applications have been test by the manufactures with the validation information for application.

Eukaryotic cell lines

Policy information about cell lines and Sex and Gender in Research

Cell line source(s)	Human HEK293T cells (CRL-3216) and mouse C2C12 myoblast cells (CRL-1772) were obtained from American Type Culture Collection (ATCC).
Authentication	Cell lines were procured from commercial source.
Mycoplasma contamination	All cell lines were tested as negative for mycoplasma contamination.
Commonly misidentified lines (See ICLAC register)	No commonly misidentified lines.

Plants

Seed stocks	Not applicable.
Novel plant genotypes	Not applicable.
Authentication	Not applicable.
